# Validation of Madecassoside Synergy Significantly Enhanced Cryptotanshinone’s Therapeutic Efficacy Against Acne Vulgaris

**DOI:** 10.3390/bioengineering12090935

**Published:** 2025-08-29

**Authors:** Yaling Guo, Xiaobin Yang, Lifeng Tang, Tao Liang, Rongshen Xiao, Qiang Liu

**Affiliations:** 1 School of Traditional Chinese Medicine, Southern Medical University, Guangzhou 510515, China; guo42110199@smu.edu.cn (Y.G.); 18520914916@163.com (T.L.); 2 State Key Laboratory of Bioactive Molecules and Druggability Assessment, Jinan University, Guangzhou 510632, China; yxbpost@stu2025.jnu.edu.cn (X.Y.); tanglifeng@tuiquan.com (L.T.); 3 Guangzhou Xika Technology Co., Ltd., Guangzhou 510220, China; xiaorongshen@tuiquan.com

**Keywords:** acne, cryptotanshinone, madecassoside, synergy, anti-inflammatory, antibacterial, zebrafish

## Abstract

Current acne therapies face major limitations, including antibiotic resistance and skin irritancy. In this study, a synergistic strategy combining cryptotanshinone and madecassoside was developed through functional complementarity. Antibacterial activity against *Cutibacterium acnes* was evaluated using minimum inhibitory concentration (MIC) and inhibition zone assays, while cytotoxicity was assessed using human keratinocytes (HaCaTs). Anti-inflammatory efficacy was quantified by measuring tumor necrosis factor-alpha (TNF-α), interleukin-1 beta (IL-1β), interleukin-6 (IL-6), and prostaglandin E_2_ (PGE_2_) in lipopolysaccharide-stimulated macrophages and a copper sulfate (CuSO_4_)-induced zebrafish inflammatory model. Systemic safety was examined in zebrafish models (developmental toxicity and sodium dodecyl sulfate-induced irritation). Finally, macroscopic severity, histopathology, and serum cytokines were used to assess an oleic acid-induced rat acne model. Cryptotanshinone inhibited *Cutibacterium acnes* (minimum inhibitory concentration = 62.5 μg/mL) but exhibited cytotoxicity (>5 μg/mL) and irritancy (≥1000 μg/mL). Madecassoside eliminated cryptotanshinone-induced cytotoxicity and reduced irritation. Importantly, the combination maintained antibacterial efficacy while synergistically enhancing anti-inflammatory effects, achieving a 94% reduction in follicular hyperkeratosis compared with 39% for cryptotanshinone alone (*p* < 0.01), alongside normalization of histopathology and cytokine levels. In conclusion, madecassoside functionally complements cryptotanshinone by neutralizing its cytotoxicity and irritancy, enabling a safe, synergistic therapy that concurrently targets antibacterial and anti-inflammatory pathways in acne pathogenesis

## 1. Introduction

Acne vulgaris is a chronic inflammatory disorder of the pilosebaceous unit; its pathogenesis is primarily driven by four key factors: follicular hyperkeratinization, sebum hyperproduction, colonization by *Cutibacterium acnes* (*C. acnes* formerly *Propionibacterium acnes*), and immune dysregulation [1,2,3]. Hyperseborrhea and follicular obstruction create a favorable niche for *C. acnes* proliferation [4]. This bacterium affects approximately 85% of adolescents globally, with 20–35% developing moderate-to-severe lesions necessitating medical intervention. Such individuals are at high risk of permanent scarring and post-inflammatory hyperpigmentation [5]. These sequelae are associated with significant psychosocial burden: meta-analyses reveal a 2.36-fold increased risk of depression and quality-of-life impairment comparable to chronic conditions such as diabetes [6].

First-line therapies include topical retinoids, antibiotics, and systemic anti-androgenic drugs. However, these are limited by antimicrobial resistance, irritation-induced barrier dysfunction, and teratogenicity in the case of isotretinoin [7]; chemical peels with α-hydroxy or salicylic acids are sometimes used but risk barrier damage when over-applied [8]. Given acne’s multifactorial pathogenesis, treatment ideally requires a multi-target strategy addressing bacterial overgrowth, inflammation, and barrier repair. Compared with synthetic drugs, bioactive compounds from medicinal plants offer superior biocompatibility, fewer side effects, and multi-target activity.

This potential is exemplified by specific natural compounds and their dermatological applications: flavonoids, which are bioactive natural polyphenolic compounds, contribute anti-inflammatory and anti-aging benefits [9]; berberine and celastrol have demonstrated therapeutic effects in the treatment of psoriasis [10,11]; moreover, mature skin formulations containing celastrol, as well as quercetin and paeonol, are already utilized in the clinical management of psoriasis and atopic dermatitis, respectively; and polygalaxanthone III, an active ingredient from *Polygala japonica* Houtt., has been shown to repair skin injury stimulated by *Malassezia* via STAT3 phosphorylated activation [12]. These examples underscore the promising synergy of natural compounds for dermatological applications. This synergy, along with the broad-spectrum antimicrobial activity (including against resistant strains), anti-inflammatory effects, sebum regulation, and barrier repair promotion offered by traditional medicinal plants such as *Salvia miltiorrhiza* (*S. miltiorrhiza*), *Scutellaria baicalensis*, *Paris polyphylla*, *Lonicera japonica*, and *Sophora flavescens*—all with favorable safety profiles [13,14,15]—further provides a promising foundation for safer and more effective acne therapeutics.

Cryptotanshinone (CTS), a lipophilic diterpene quinone from *S. miltiorrhiza*, exhibits antioxidant, antimicrobial, and anti-inflammatory properties. It reduces sebum secretion, modulates follicular keratinization, and suppresses inflammatory signalling [16,17,18]. Our laboratory previously enhanced CTS solubility and bioavailability via nanoliposomes and micelles, demonstrating strong antimicrobial and anti-inflammatory effects in in vitro assays and *C. acnes*-induced BALB/c nude mouse models [19,20]. CTS demonstrates potent activity against *C. acnes*, with a minimum inhibitory concentration (MIC) of 62.5 μg/mL, and effectively suppresses Toll-like receptor 2 (TLR_2_)/nuclear factor kappa-B (NF-κB)-mediated production of pro-inflammatory cytokines interleukin-6 (IL-6) and interleukin-1β (IL-1β) [21]. Nonetheless, CTS faces major translational barriers, including cytotoxicity, irritancy, poor solubility, and limited dermal penetration.

Madecassoside (MC), a triterpenoid saponin from *Centella asiatica*, exhibits broad anti-inflammatory, antioxidant, and wound-healing properties with excellent biosafety. It promotes fibroblast regeneration and extracellular matrix remodelling, aligning well with acne pathophysiology by supporting barrier repair and mitigating scarring [22]. However, MC is associated with negligible antibacterial activity and poor dermal penetration due to its high polarity and large molecular weight [23,24,25].

Inspired by the principle of functional complementarity [26], this study aimed to establish a synergistic anti-acne strategy by combining CTS and MC. We hypothesized that CTS and MC could act synergistically: CTS providing potent antibacterial and anti-inflammatory activity, and MC mitigating toxicity while enhancing tissue repair. Therefore, we systematically evaluated CTS, MC, and their combination across in vitro cell models, zebrafish assays, and a rat acne model to assess pharmacological synergy, safety, and formulation feasibility.

## 2. Materials and Methods

### 2.1. Ethics Statement

All of the experimental procedures were conducted in compliance with the ethical guidelines provided by the Ministry of Science and Technology of China and received formal approval from the Ethics Committee of Southern Medical University (approval No. SMUL202404013). All zebrafish experiments complied with the Organisation for Economic Co-operation and Development Test Guidelines (TG203, TG236).

### 2.2. Cell Culture

HaCaT cells (Chinese Academy of Sciences) were cultured in a 75 cm^2^ flask under standard conditions (37 °C, 5% CO_2_, 100% humidity) in DMEM (Gibco BRL Co., Ltd., Grand Island, NY, USA) medium supplemented with 10% (*v*/*v*) FBS (Gibco BRL Co., Ltd., Grand Island, NY, USA) and 1% (*v*/*v*) penicillin–streptomycin. RAW 264.7 murine macrophages (Chinese Academy of Sciences) were cultured in 75 cm^2^ flasks under standard sterile conditions (37 °C, 5% CO_2_, 100% humidity) using DMEM-H (High Glucose, Gibco BRL Co., Ltd.) supplemented with 10% (*v*/*v*) fetal bovine serum (FBS) and 1% (*v*/*v*) penicillin–streptomycin.

### 2.3. Cytotoxicity Test

The cytotoxicity of CTS, MC, and their combination in HaCaT cells was evaluated using the Cell Counting Kit-8 (CCK-8) assay (Beijing Solarbio Science Technology Co., Ltd., Beijing China). Cells were seeded in 96-well plates at a density of 1 × 10^4^ cells per well and allowed to adhere for 24 h. Subsequently, cells were treated with a range of concentrations of the compounds, as detailed in Table 1. The control group contained 1% dipropylene glycol (DPG, Dow Chemical (Shanghai) Co., Ltd., Shanghai, China). After a 24 h incubation period, the medium was replaced with fresh medium containing 10% CCK-8 solution. After a 1 h incubation, the absorbance at 450 nm was measured using a microplate reader (Thermo Fisher Scientific, Waltham, MA, USA). Cell viability was calculated relative to the untreated control group. All experiments were performed in triplicate.

### 2.4. ELISA Analysis of Inflammatory Cytokines

#### 2.4.1. Cell Stimulation and Supernatant Collection

RAW 264.7 macrophages were seeded into six-well plates at a density of 1 × 10^6^ cells per well. After 24 h, cells were pretreated for 2 h with the compounds or controls listed in Table 2. Following pretreatment, all groups except the blank control were stimulated with 1 μg/mL of lipopolysaccharide (LPS) for 24 h to induce an inflammatory response. Cell culture supernatants were then collected, centrifuged (3000 × rpm, 10 min, 4 °C) to remove debris, and stored at −80 °C until analysis.

#### 2.4.2. ELISA Quantification of Inflammatory Factors

Concentrations of TNF-α, IL-1β, IL-6, and PGE_2_ in the supernatants were quantified using commercial ELISA kits (Jiangsu Meimian Industrial Co., Ltd., Yancheng, China) according to the manufacturers’ protocols. Blank-well optical density (OD_450_) readings were subtracted from sample readings to obtain corrected OD values. Sample concentrations were determined by interpolating the corrected OD values into their respective standard curves. All samples and standards were analyzed in duplicate.

### 2.5. Minimum Inhibition Concentration

*C. acnes* (Guangdong Microbial Culture Collection Center) was cultured on reinforced clostridial medium (RCM, Shenzhen Yibaishun Technology Co., Ltd., Shenzhen, China) at 37 °C under anaerobic conditions (anaerobic jar with gas-generating sachets). The MIC of CTS, MC, and MC-CTS against *C. acnes* was determined via broth microdilution. Bacterial suspensions were prepared by adjusting colonies from RCM agar to 0.5 McFarland turbidity in sterile saline. Serial two-fold dilutions of compounds (2–1024×) were prepared in 500 μL of RCM broth across 11 tubes, with the 12th tube serving as a growth control (no drug). Each tube was inoculated with 10 μL bacterial suspension (final inoculum ~5 × 10^5^ CFU/mL) and incubated anaerobically at 37 °C for 24 h. The MIC is defined as the lowest concentration that completely inhibits visible growth. Experiments were performed in triplicate.

### 2.6. Inhibition Zone

CTS (purity > 98%, Baoji Herbest Bio-Tech Co., Ltd., Baoji, China) was dissolved in DPG to generate serial concentrations (0–10 mg/mL). MC (purity > 95%, Guangxi Changzhou Natural Pharmaceutical Co., Ltd., Nanning, China) was diluted in deionized water across an equivalent concentration range (0–10 mg/mL). The MC-CTS complex was prepared in a DPG/water (1:1 *v*/*v*) solvent system at matching concentrations (0–10 mg/mL). The positive control, Quaternium-73 (Q-73), was formulated at 1 mg/mL (0.1% *w*/*v*) in DPG.

Lawns of *C. acnes* were established on RCM agar plates, onto which six-mm sterile paper disks were aseptically placed. Three experimental sets were implemented: (1) CTS dose–response: disks received 10 μL of vehicle (DPG), positive control (Q-73, 1 mg/mL), high-dose CTS (3 mg/mL), or low-dose CTS (1 mg/mL); (2) MC dose-response: the same setup was used with MC; (3) combination assessment: disks loaded with DPG, Quaternium-73, CTS (1 mg/mL), and MC-CTS (MC 1 mg/mL + CTS 1 mg/mL). Plates were incubated anaerobically at 37 °C for 24 h and 48 h, and inhibition zone diameters (including disk) were measured. Triplicate biological replicates were performed.

### 2.7. Zebrafish

#### 2.7.1. Zebrafish Maintenance and Embryo Production

Adult zebrafish (*Danio rerio*, National Zebrafish Resource Center) of the wild-type (WT) AB strain and transgenic *Tg*(*lyz*:*DsRed*) strain were maintained in a recirculating aquaculture system (Shanghai Haisheng Biological Equipment Co., Ltd., Shanghai, China) under controlled conditions: a 14 h light/10 h dark photoperiod, water temperature of 26.0–29.0 °C, pH 7.0–8.0, and conductivity 500–550 μS/cm. Fish were fed live brine shrimp (*Artemia* nauplii) ad libitum twice daily.

For embryo production, breeding tanks were established with male/female separators the evening prior to fertilization, at a ratio of 1:1. Separators were removed the following morning to initiate spawning. Fertilized eggs were collected via bottom-mounted mesh grids within 1 h post-spawning, selected for viability, and incubated in E3 embryonic medium (5 mM NaCl, 0.17 mM KCl, 0.33 mM CaCl_2_, 0.33 mM MgSO_4_, methylene blue, 0.1% (*m*/*m*), pH 7.2) at 28.0–29.0 °C. Nonviable embryos were routinely removed during incubation.

#### 2.7.2. Toxicity and Irritability Assessment in Zebrafish

##### 24 h Acute Toxicity Test

Healthy 24 hpf WT AB embryos and 3 dpf *Tg*(*lyz*:*DsRed*) embryos were exposed to CTS (5–320 μg/mL) and/or MC (25–1600 μg/mL) in 96-well plates (1 embryo per well, 12 replicates, 200 μL E3 medium). Control embryos were exposed to E3 medium with 1% DMSO. Survival and teratogenicity (absence of heartbeat indicating death) rates were assessed at 48 hpf (WT AB) or 4 dpf (*Tg*(*lyz*:*DsRed*)). Experiments were independently repeated in triplicate. Survival (%) = (number of surviving embryos/total number of exposed embryos) × 100%

##### Phenotype Recording

Healthy WT AB zebrafish embryos (24 hpf) and *Tg*(*lyz*:*DsRed*) zebrafish embryos (3 dpf) were collected, randomly grouped (20 per group), and exposed in 24-well plates (2 mL solution per well) to 1% DMSO (control), CTS (5–320 μg/mL), MC (25–1600 μg/mL), or combinations thereof. After 24 h of exposure, individual phenotypes were imaged using a stereo fluorescence microscope (Leica MZ10F, Leica Microsystems, Wetzlar, Germany).

#### 2.7.3. Sodium dodecyl sulfate (SDS)-Induced Zebrafish Embryos Irritancy Model (Self-Rotation Behavior)

To evaluate soothing effects, 24 hpf WT AB embryos (n = 10/well, triplicate) were placed in 96-well plates. Groups included: control (200 μL E3 medium/well), model (500 μM SDS), positive control (500 μM SDS + 1 mM dipotassium glycyrrhizinate, DG), MC (500 μM SDS + 0.05–10 mg/mL), CTS (500 μM SDS + 0.05–2 mg/mL), or CTS-MC combinations at different ratios.

After 1 h of pretreatment and 15 min of SDS-Induced modelling, the self-rotation frequency of embryos within 30 s was quantified.

The spin inhibition rate (%) was calculated as follows: [(Mean rotations _Model group_ − Mean rotations _Treatment group_)/Mean rotations _Model group_] × 100%. Data were presented as mean ± standard error of the mean (SEM). This approach facilitated systematic screening for optimal combinatorial formulation efficacy.

#### 2.7.4. High-Dose CTS-Induced Zebrafish Embryos Irritability Model

Building directly upon the SDS-induced model (Section 2.7.3), this assay utilized high-dose CTS (1 mg/mL) as the irritant stimulus instead of SDS. The core methodology (24 hpf WT AB strain embryos; n = 10/well, triplicates; 200 µL E3 medium/well; 28 ± 1 °C incubation; quantification of 30 s embryos self-rotation frequency via stereo fluorescence microscopy) remained identical.

#### 2.7.5. Pharmacological Attenuation of CuSO_4_-Induced Neutrophil Migration

Acute inflammation was induced in 3 dpf *Tg*(*lyz*:*DsRed*) zebrafish embryos (24-well plates,2 mL E3 medium/well, n = 20 per group) by exposure to 20 μM CuSO_4_.

Meanwhile, medicine intervention groups were treated with positive drugs: dexamethasone (Dex, 5 μg/mL) and different concentrations of CTS (0–80 μg/mL), MC (0–800 μg/mL), and CTS-MC at varying concentration ratios; zebrafish were immobilized in 3% (*w*/*v*) methylcellulose-coated slides. Compounds were administered in a gradient dosing regimen at 5 min intervals. After 2 h of co-incubation with CuSO_4_ and treatments, neutrophil migration to the lateral line neuromasts was quantified using a stereo fluorescence microscope (Leica MZ10F, Germany). Successful model induction was confirmed by significant neutrophil infiltration into the dorsal neural tube (control baseline: 4.73 ± 0.48 cells/embryo).

Neutrophil Inhibition Rate Calculation:

Inhibition rate (%) = (C − T)/C × 100%

where C = the mean neutrophil count in the model group; T = the mean count in the treatment group.

### 2.8. Establishment of Rat Acne Model and Therapeutic Intervention

#### 2.8.1. Formulation Development and Stability Assessment

##### Solvent Selection

DPG was selected as the optimal solvent for CTS due to its high solubility (1:20, *w*/*w*, 90 ± 5 °C), a favorable safety profile, desirable skin feel, and low cost. Alternatives exhibited inferior solubility (e.g., butylene glycol and diisopropyl adipate, exhibited poor solubility (1:50, *w*/*w*) and precipitated at −15 °C) or higher cost (pentylene glycol and dimethyl isosorbide, approximately 10 times the cost of DPG). Glycerin was excluded as it induced adhesion; propylene glycol had potential safety concerns (EWG rating = 3, Environmental Working Group).

##### Emulsification process

Formulations (600 g batch, pH 6.0–6.5 in 10% aqueous solution, viscosity 60–70 kPa·s) were prepared as follows:

CTS premix: 0.15 g of CTS in 18 g of DPG (85 ± 5 °C).

Preservative premix: 3 g of hydroxyacetophenone + 3 g of 1,2-hexanediol in 12 g of DPG (60–70 °C).

Aqueous phase: xanthan gum (0.6 g) and carbomer 940 (Lubrizol, USA, 1.8 g) dispersed in deionized water, heated to 85 ± 5 °C, with disodium EDTA (0.18 g), 85 ± 5 °C, homogenized (IKA T25, Germany, 5 min).

Oil phase: Montanov™ 68 (cetearyl glucoside/cetearyl alcohol, 9.0 g, SEPPIC S.A., La Garenne Colombes, Paris, France), Emercol^®^ C16-18 30:70 MY (cetearyl alcohol, 7.2 g), SP ARIACEL 170 MBAI-PA-(SG) (glyceryl stearate/PEG-100 stearate, 6.0 g, Croda International plc, Snaith, UK), GTCC (caprylic/capric triglyceride, 18.0 g, Croda International plc, Snaith, UK), isononyl isononanoate (12.0 g), dimethicone (12.0 g), squalane (9.0 g), 85 ± 5 °C.

The oil phase was added to the aqueous phase, homogenized (20,000 rpm, 5 min), and followed by the addition of Simulgel™ EG (SEPPIC S.A., La Garenne Colombes, Paris, France, 1.8 g) and sodium surfactin (Kaneka Corporation, Osaka, Japan, 0.3 g) and re-homogenization. Preservative premix was added at 60 °C, pH was adjusted with arginine at 45 °C, and the mixture was cooled to 25 °C.

Formulations included blank base, MC (1 mg/g), CTS (0.25 mg/g), and MC + CTS (1 mg/g + 0.25 mg/g), (200 g batches).

##### Formulation Stability Assessment

Stability was evaluated over scheduled intervals (3, 7, 15, 30, 60, and 90 days) under a range of stress conditions. These included thermal stress tests to simulate extreme seasonal variations, specifically, heating at 45 ± 1 °C, freezing at −15 ± 1 °C, and cyclic testing between these two temperatures. Additionally, stability was assessed following centrifugation (3000 rpm, 30 min) and UV exposure for photostability. Concurrently, key physicochemical parameters, namely pH and viscosity, were also monitored throughout the study.

At each time point, formulations were examined for changes in appearance (color, opacity, phase separation, precipitation), pH, and viscosity. Viscosity was measured to detect early signs of network collapse or coalescence, while pH stability was critical for maintaining both preservative efficacy and CTS solubility. Where applicable, data were expressed as mean ± SEM to account for variability across replicates. This comprehensive stability assessment ensured that the formulations maintained consistent physicochemical properties throughout the experimental period, thereby guaranteeing reliable dosing and performance during in vivo therapeutic evaluation in the rat acne model.

#### 2.8.2. Oleic Acid-Induced Rat Acne Model and Therapeutic Intervention

A total of fifty-six male Sprague-Dawley (SD) rats (200–230 g; SCXK(Yue)2021-0041), sourced from the Experimental Animal Center of Southern Medical University, were utilized for the acne model.

After acclimatization for 3 days, these rats were randomly assigned into 7 groups (n = 8) using a random number table to ensure unbiased allocation: (1) naive control, (2) untreated model, (3) positive control (KAC gel), (4) vehicle control (base cream), (5) MC monotherapy, (6) CTS monotherapy, and (7) MC-CTS combination therapy. Animals were housed under controlled conditions (temperature 22 ± 2 °C, relative humidity 55 ± 5%, 12 h light/12 h dark cycle), with ad libitum access to standard chow and water.

After anesthesia and dorsal depilation (3 × 3 cm area), all except the naive controls received daily topical oleic acid (100%, 1 mL/rat, 14 days) to induce acne-like lesions, characterized by follicular hyperkeratosis, erythema, and inflammatory infiltration. Body weight was monitored daily. Following induction, groups 3–7 received once-daily topical treatments (1 g, 3 days): Group 3 received commercial anti-acne KAC gel, Group 4 received the base cream, Group 5 received MC cream (1 mg/g), Group 6 received CTS cream (0.25 mg/g), and Group 7 received the MC-CTS combination cream (1 mg/g + 0.25 mg/g). Group 2 remained untreated. Dorsal skin condition and body weight were recorded before sacrifice.

Skin lesions were assessed on days 0, 5, 10, 12, and 14 using standardized criteria. Erythema, scaling, and follicular plugging were each scored on a 0–3 scale (0 = none, 1 = mild, 2 = moderate, 3 = severe). The total acne severity score was the sum of these subscores. Standardized high-definition photographs were taken under consistent lighting.

### 2.9. Histopathological Analysis

On Day 14, following euthanasia, a 1.0 × 1.0 cm section of dorsal skin was aseptically collected. Tissues were fixed in 4% paraformaldehyde (PFA)/phosphate-buffered saline (PBS) at 4 °C for 24–48 h, rinsed in PBS, transferred to 70% ethanol, and labeled with group identifiers. All specimens were shipped under cold-chain conditions to Wuhan Bioqiandu Technology Co., Ltd. (Wuhan, China) for paraffin embedding, microtome sectioning (5 μm), and hematoxylin–eosin (HE) staining. Digital images were captured using a KF-PRO-005 Digital slice scanner (Jiangfeng, Ningbo, China), with three random fields per slide assessed for dermal histopathological alterations (e.g., hyperkeratosis, inflammatory infiltration, and follicular dilation) by an investigator blinded to group assignments.

### 2.10. Serum Cytokine Quantification

Blood samples were collected via cardiac puncture, and serum was isolated by centrifugation. The levels of inflammatory cytokines (TNF-*α*, IL-1*β*, IL-6, and PGE_2_) in the serum were quantified using specific commercial rat ELISA kits (Jiangsu Meimian Industrial Co., Ltd., Jiangsu, China) according to the manufacturer’s instructions.

### 2.11. Data Analysis

All experiments were performed with at least three independent replicates.Results were presented as mean ± SEM. Statistical analyses were performed using GraphPad Prism software (version 10.1.2, GraphPad Software, San Diego, CA, USA). All data were analyzed using one-way ANOVA or Student’s *t*-test, while IC_50_/LC_50_ values were calculated by nonlinear regression, with *p* < 0.05 considered statistically significant.

## 3. Results

### 3.1. The Pharmacological Effects of CTS

#### 3.1.1. Antibacterial Activity of CTS

The in vitro antibacterial efficacy of CTS against *C. acnes* was quantitatively assessed using MIC and zone of inhibition assays. MIC analysis revealed significant growth inhibition at CTS concentrations ≥ 62.50 μg/mL. Although literature reports indicate that the positive control Q-73 exhibits superior potency at considerably lower MIC values (0.00002–0.00004%), CTS demonstrated even more pronounced efficacy in the agar diffusion assay.

**Figure 1:**
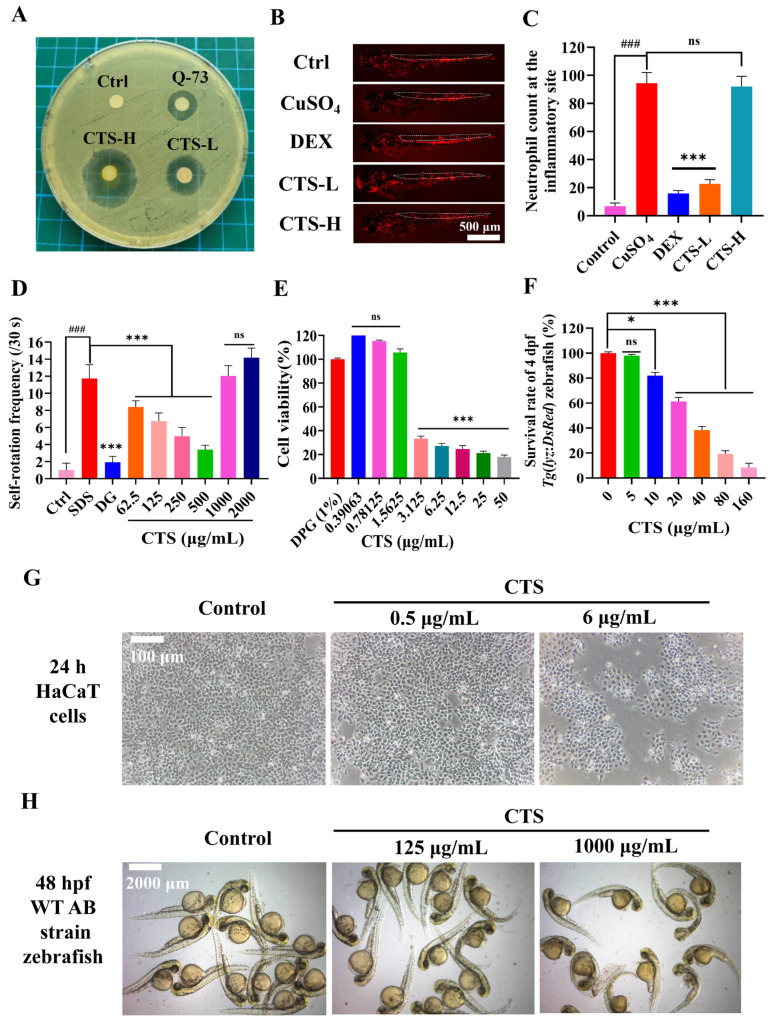
Antimicrobial, anti-inflammatory, and safety profile of CTS. (**A**) *C. acnes* inhibition zone (CTS-H 3.0 mg/mL; CTS-L: 1.0 mg/mL); Ctrl: DPG solvent control; Q-73 (Quaternium-73): positive control (1.0 mg/mL) (n = 3). (**B**,**C**) Neutrophil distribution in the inflammation model of 3 dpf *Tg*(*lyz*:*DsRed*) zebrafish induced by 20 µM CuSO_4._ (n = 20); Ctrl: E3 medium; Dex: 5 µg/mL; CTS-L: 40 µg/mL; CTS-H: 200 µg/mL. (**D**) SDS (500 μM)-induced self-rotation frequency at 24 hpf embryos of WT AB strain zebrafish (n = 16); DG: 1 mM; CTS: 62.5–2000 μg/mL. (**E**) Cell viability of HaCaT keratinocytes following 24-h incubation with CTS at different concentrations (n = 6). (**F**) Survival rates of 3 dpf *Tg*(*lyz*:*DsRed*) zebrafish following a 24-h exposure to different concentrations of CTS (n = 12). (**G**) Representative images of HaCaT cells. (**H**) Representative images of the teratogenic effects of WT AB strain zebrafish at 48 hpf. Data were presented as mean ± SEM. “###” denoted *p* < 0.001 compared with the blank control. Wherever included, comparisons with the model group were indicated as n.s. (non-significant, *p* > 0.05), * *p* < 0.05, and *** *p* < 0.001.

Comparative inhibition zone testing at equivalent concentrations (1 mg/mL) demonstrated substantially larger zones for CTS (15.8 ± 0.3 mm) versus Q-73 (11.2 ± 0.7 mm). High-concentration CTS (3 mg/mL) further increased efficacy (19.3 ± 0.6 mm). These results indicate that CTS generates approximately 1.4–1.5-fold larger inhibitory zones than Q-73 against *C. acnes* at equivalent concentrations, confirming superior bacteriostatic activity. DPG controls demonstrated no zones of inhibition (0 mm), confirming the absence of antibacterial activity and validating the integrity of the experimental conditions (Figure 1A).

#### 3.1.2. CuSO_4_-Induced Neutrophil Migration in 3 dpf *Tg*(*lyz*:*DsRed*) Zebrafish

In the CuSO_4_-induced zebrafish inflammation model, the model group exhibited a significant inflammatory response characterized by markedly increased infiltration of inflammatory cells (predominantly neutrophils), indicating robust neutrophil migration to the injury site. The administration of CTS, however, substantially reduced the number of recruited inflammatory cells at specific concentrations. Notably, this inhibitory effect of CTS-L (40 μg/mL, *p* < 0.001 vs. CuSO_4_) on neutrophil migration was comparable to that observed with the positive control drug DEX. These results demonstrated that CTS, within its safe dosage range, significantly ameliorated CuSO_4_-induced inflammation by effectively suppressing neutrophil recruitment in zebrafish, thereby confirming its potent anti-inflammatory activity. Conversely, higher CTS concentrations (200 μg/mL, *p* > 0.05 vs. CuSO_4_) failed to exhibit a significant anti-inflammatory effect. This distinct pharmacological profile, characterized by anti-inflammatory efficacy with a narrow therapeutic window, poses significant challenges for formulators in pharmaceutical applications (Figure 1B,C).

#### 3.1.3. SDS-Induced Self-Rotation Behavior in 24 hpf embryos of WT AB Zebrafish

An assessment of the soothing effect (measured by relative self-rotation counts) revealed that SDS stimulation significantly increased self-rotation frequency, indicating stress-induced discomfort. This behavior was mitigated by the positive control DG (1 µM). CTS treatment exerted a pronounced dose-dependent effect on this parameter:

Low-to-mid concentrations (62.5–500 μg/mL): CTS treatment resulted in a dose-dependent decrease in self-rotation frequency. Increasing CTS concentrations within this range progressively reduced rotation counts (*p* < 0.001 vs. SDS), demonstrating its capacity to alleviate SDS-induced stress or discomfort.

High concentrations (1000–2000 μg/mL): CTS paradoxically increased self-rotation frequency relative to the model group. This reversal suggests that CTS not only loses its soothing efficacy at high doses but may induce additional irritation or adverse effects, exacerbating stress-related behavior (Figure 1D).

#### 3.1.4. CTS Cytotoxicity in Human HaCaT Keratinocytes

In vitro assessment demonstrated the concentration-dependent cytotoxicity of CTS in human skin keratinocytes. While 1.5 μg/mL CTS maintained 100% cell viability (comparable to control, *p* > 0.05), significant toxicity emerged at >3 μg/mL: 3.125 μg/mL reduced viability to 40% (*p* < 0.001 vs. control), and 12.5 μg/mL caused substantial damage (30% viability; *p* < 0.001 vs. control). Morphological analysis confirmed this toxicity threshold, with 16 μg/mL CTS inducing characteristic apoptotic/necrotic features, including cellular contraction and fragmentation. Thus, CTS cytoxicity threshold is ~3 μg/mL (Figure 1E,G).

#### 3.1.5. Developmental Toxicity in the Zebrafish Model

In vivo evaluation revealed concentration-dependent systemic toxicity in zebrafish embryos. Survival rates of *Tg*(*lyz*:*DsRed*) zebrafish at 4 dpf demonstrated no adverse effects (CTS ≤ 5 μg/mL; 100% survival, n.s. vs. control). Mild toxicity was observed at 10 μg/mL (80% survival), while 80 μg/mL caused severe lethality (20% survival; *p* < 0.001 vs. control). Surviving embryos at 80 μg/mL exhibited critical developmental abnormalities, including spinal curvature and edema. The maximum tolerated concentration was <10 μg/mL, with teratogenic effects manifested at sublethal doses. Despite exhibiting higher tolerance to CTS than 4 dpf *Tg*(*lyz*:*DsRed*) zebrafish, WT AB zebrafish at 48 hpf still demonstrated obvious teratogenic effects at a high concentration of 1000 μg/mL (Figure 1F,H).

### 3.2. MC Influences the Safety Profile of CTS

#### 3.2.1. MC Attenuates CTS Cytotoxicity in HaCaT Cells

**Figure 2:**
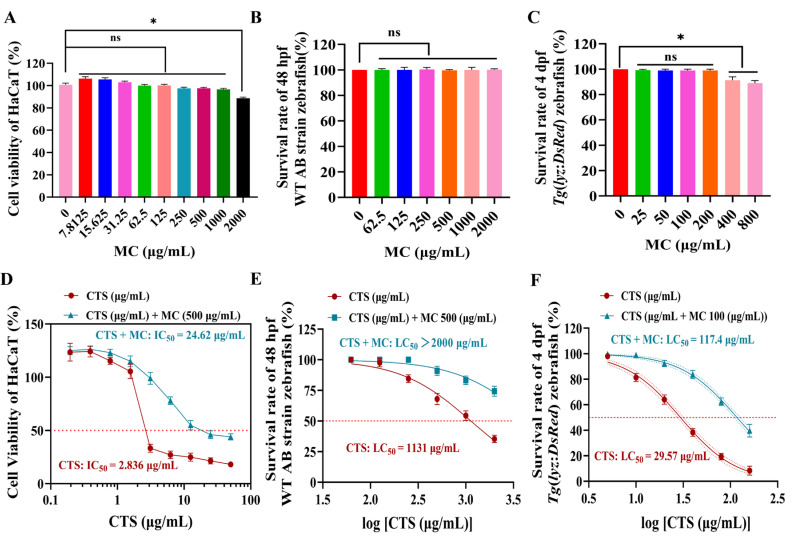
By mitigating high-dose CTS toxicity, MC significantly improved the safety profile of CTS. (**A**,**D**) (**A**) Cell viability of HaCaT keratinocytes following 24-h incubation with MC at different concentrations (n = 6); (**D**) The cytotoxicity threshold (IC_50_) in HaCaT keratinocytes (CTS vs. CTS-MC). (**B**,**E**) Survival rate and LC_50_ of WT AB strain zebrafish at 48 hpf (n = 12): (**B**, MC) and (**E**, CTS vs. CTS-MC). (**C**,**F**) Survival rate and LC_50_ of *Tg*(*lyz*:*DsRed*) zebrafish at 4 dpf (n = 12): (**C**, MC) and (**F**, CTS vs. CTS-MC). Data were presented as mean ± SEM. Wherever included, comparisons with the control group were indicated as n.s. (non-significant, *p* > 0.05), * *p* < 0.05.

MC (7.8125–1000 μg/mL) demonstrated no significant growth inhibition in HaCaT cells after 24 h. Significant inhibition only occurred at 2000 μg/mL (*p* < 0.05 vs. control) (Figure 2A). Remarkably, co-treatment with 500 μg/mL MC significantly improved survival in cells exposed to CTS. The Half-Maximal Inhibitory Concentration (IC_50_) of CTS alone was 2.836 μg/mL, but when combined with 500 μg/mL MC, the IC_50_ increased to 24.62 μg/mL, indicating an 8.7-fold reduction in CTS cytotoxicity (*p* < 0.001 vs. CTS) (Figure 2D).

#### 3.2.2. MC Modulated CTS-Induced Toxicity in Zebrafish Embryos

MC exhibited no significant effect on survival in 48 hpf WT AB strain zebrafish embryos at tested concentrations (62.5–2000 μg/mL) (100% survival, n.s. vs. control) (Figure 2B). In contrast, CTS alone demonstrated potent toxicity in 24 hpf WT AB zebrafish, with an Lethal Concentration 50 (LC_50_) of 1131 μg/mL. Strikingly, co-exposure to 500 μg/mL MC and CTS markedly reduced CTS toxicity, elevating the LC_50_ to > 2000 μg/mL (*p* < 0.001 vs. CTS) (Figure 2E). This represented a > 77% increase in the half-maximal lethal concentration of CTS, indicating that MC can antagonize the acute toxicity induced by high-dose CTS significantly.

MC (25–200 μg/mL) demonstrated no significant impact on survival in 4 dpf *Tg*(*lyz*:*DsRed*) strain zebrafish embryos (n.s. vs. control). However, at higher concentrations (400 and 800 μg/mL), MC induced significant growth inhibition (*p* < 0.05 vs. control) (Figure 2C).

Exposure to CTS alone in 4 dpf *Tg*(*lyz*:*DsRed*) zebrafish yielded an LC_50_ of 29.57 μg/mL. Strikingly, co-treatment with 500 μg/mL MC substantially attenuated CTS toxicity, elevating the LC_50_ to 117.4 μg/mL. This represented a 4-fold increase in the half-maximal lethal concentration of CTS, demonstrating potent toxicity antagonism by MC (*p* < 0.001 vs. CTS) (Figure 2F).

#### 3.2.3. MC Reduced CTS-Induced Morphological Abnormalities in Zebrafish at Multiple Developmental Stages

**Figure 3:**
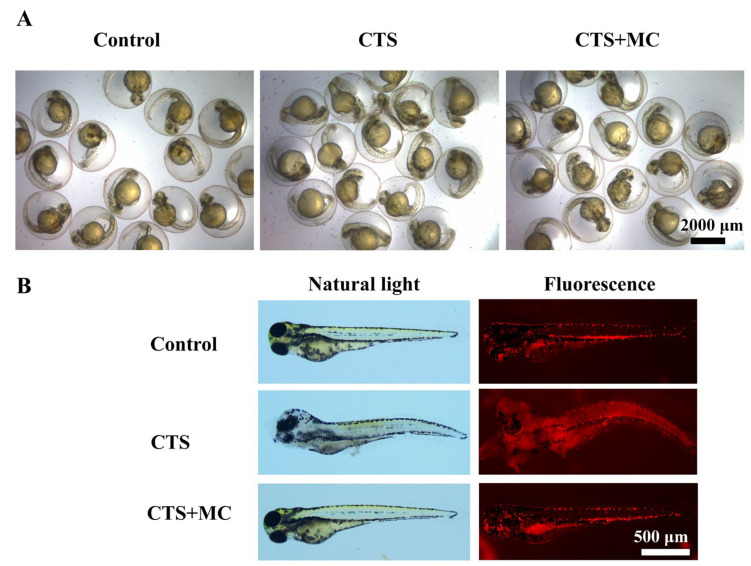
By mitigating high-dose CTS toxicity, MC significantly improved the safety profile of CTS. (**A**) Representative images of WT AB zebrafish embryos at 48 hpf. (**B**); Representative images of *Tg*(*lyz*:*DsRed*) zebrafish embryos at 4 dpf.

Acute toxicity assessment in 24 hpf WT AB zebrafish embryos (48 hpf; safety threshold: CTS ≤ 250 μg/mL) revealed normal development, including a regular heartbeat, intact yolk sac, and defined structures, in the control, CTS (≤250 μg/mL), and CTS + MC (≤250 μg/mL) groups (Figure 1H and Figure 3A). However, exposure to 1000 μg/mL of CTS induced significant abnormalities, characterized by an irregular heartbeat, yolk sac atrophy, and developmental arrest, which correlated with moderate toxicity at 50% survival. 

Evaluation in 4 dpf *Tg*(*lyz*:*DsRed*) zebrafish embryos (safety threshold: CTS ≤ 5 μg/mL) demonstrated a normal phenotype with regular heartbeat and yolk sac in the control, CTS (≤5 μg/mL), and CTS + MC (≤5 μg/mL) group. The control exhibited a minimal baseline number of *DsRed* neutrophils. Exposure to 40 μg/mL of CTS caused severe teratogenic effects, including tail curvature, pericardial edema, impaired yolk resorption, and a marked reduction in neutrophil counts, and abnormal distribution (indicating neutrophil dysregulation), which was associated with severe toxicity (40% survival). Co-treatment with MC (100 μg/mL) preserved normal brightfield morphology despite CTS exposure (40 μg/mL), yet displayed an altered neutrophil distribution compared to the CTS-only group. This demonstrated that MC mitigated CTS-induced neutrophil dysregulation and damage via molecular modulation (Figure 3B).

### 3.3. Antibacterial, Anti-Inflammatory, and Soothing Effects of the MC-CTS Combination

#### 3.3.1. Antibacterial Activity of the CTS-MC Combination

**Figure 4:**
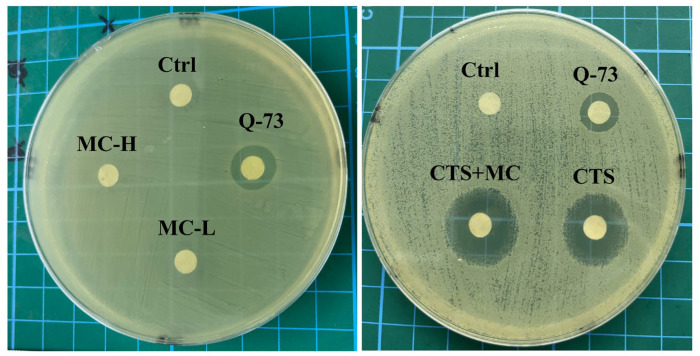
The antimicrobial activity of MC and MC + CTS.

Both high-dose (3 mg/mL) and low-dose (1 mg/mL) of MC groups (MC-H and MC-L) exhibited no intrinsic antibacterial activity, as demonstrated by confluent bacterial growth devoid of inhibition zones. Crucially, the combination of CTS-MC (1 mg/mL + 1 mg/mL) maintained bactericidal potency equivalent to that of CTS monotherapy. Quantitative assessment demonstrated a comparable reduction in colony density in the combination and CTS alone groups, with no statistically significant difference (*p >* 0.05; CTS vs. CTS-MC) (Figure 4).

#### 3.3.2. Soothing Effects

##### SDS-Induced Self-Rotation Behavior in Zebrafish

**Figure 5:**
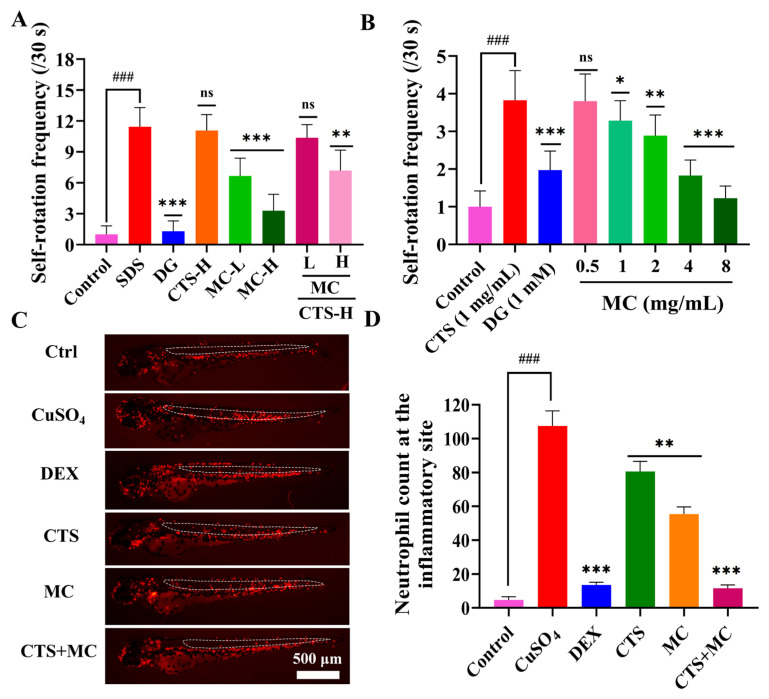
The complementary biological effects of MC and CTS in acne-related models. (**A**,**B**) Relative self-rotation frequency in 24 hpf WT AB strain zebrafish (n = 16); DG: 1 mM; (**A**) SDS (500 μM)-induced self-rotation frequency/(30 s); (**B**) High-dose of CTS (1 mg/mL)-induced self-rotation frequency/(30 s). (**C**,**D**) Neutrophil distribution in the inflammation model of 3 dpf *Tg*(*lyz*:*DsRed*) zebrafish induced by 20 µM CuSO_4_ (n = 20); Ctrl: E3 medium; Dex: 5 µg/mL; CTS: 20 µg/mL; MC: 200 µg/mL; CTS + MC: (20 µg/mL + 200 µg/mL). Data were presented as mean ± SEM. “###” denoted *p* < 0.001 compared with the blank control. Wherever included, comparisons with the model group were indicated as n.s. (non-significant, *p* > 0.05), * *p* < 0.05, ** *p* < 0.01, and *** *p* < 0.001.

To evaluate the anti-irritant effects of CTS and MC co-administration in 24 hpf WT AB zebrafish, doses of 0–2000 μg/mL for each compound were tested. The results demonstrated that CTS at doses below 500 μg/mL exhibited intrinsic soothing activity, with enhanced efficacy when combined with MC (500–2000 μg/mL). Conversely, CTS at 1000 μg/mL induced significant irritation, increasing embryo rotations to 11.07 ± 0.40 (comparable to the model group: 11.44 ± 0.63; *p* < 0.001 vs. control), and resulting in only a 3.24% reduction in relative self-rotation (n.s. vs. SDS). The co-administration of high-dose CTS (1000 μg/mL) with lower MC (500 μg/mL) partially mitigated the irritation effect (19.09% inhibition; *p* < 0.01 vs. SDS), though rotational counts remained elevated (10.37 ± 0.33). MC monotherapy demonstrated dose-dependent efficacy (500 μg/mL: 41.75%; 1000 μg/mL: 71.2% inhibition; *p* < 0.001 vs. SDS), while the positive control (1 µM DG) achieved 88.67% inhibition (*p* < 0.001 vs. SDS). These findings indicate synergistic anti-inflammatory effects at low CTS doses (<500 μg/mL) with MC, whereas high-dose CTS (≥1000 μg/mL) induces irritation that MC only partially alleviates (Figure 5A).

##### High-Concentration CTS-Induced Irritancy Model

A series of gradient doses of MC was applied to mitigate the stimulatory effects induced by high-dose CTS (1 mg/mL) substitution of SDS in the model system. The results demonstrated that MC exhibited a dose-dependent detoxification effect against CTS. At low concentrations (0.5 mg/mL), MC demonstrated minimal improvement in the relative spin frequency of 24 hpf WT AB zebrafish embryos that was not statistically significant (*p* > 0.05 vs. CTS). In contrast, a concentration of 1 to 2 mg/mL resulted in a statistically significant reduction of the relative spin frequency (*p* < 0.05 vs. CTS), while concentrations ranging from 4 to 8 mg/mL produced a highly significant improvement (*p* < 0.001 vs. CTS) (Figure 5B).

#### 3.3.3. CuSO_4_-Induced Neutrophil Migration

The combinatorial formulation of CTS (20 μg/mL) and MC (200 μg/mL), identified through optimization screening (CTS: 0–80 μg/mL; MC: 0–800 μg/mL), demonstrated superior anti-inflammatory efficacy in the CuSO_4_-induced 3 dpf *Tg*(*lyz*:*DsRed*) zebrafish neutrophilic inflammation model. Neutrophil quantification revealed profound infiltration in the model group (103.2 ± 2.18 cells/tail) compared to untreated controls (4.73 ± 0.48 cells/tail). Dexamethasone (5 μg/mL) significantly attenuated migration (13.93 ± 0.42 cells/tail; 87.75% reduction vs. the model, *p* < 0.001). Monotherapy with CTS (20 μg/mL) or MC (200 μg/mL) yielded moderate reductions (80.6 ± 1.54 cells/tail, 22.21% inhibition, *p* < 0.01; and 55.4 ± 1.10 cells/tail, 46.99% inhibition, *p* < 0.01, vs. the model, respectively). Notably, the CTS-MC combination almost completely suppressed neutrophil migration (11.6 ± 0.62 cells/tail), representing a 90.04% reduction versus the model group (*p* < 0.001), equivalent to DEX and closely approaching baseline control levels (Figure 5C,D), confirming marked synergistic anti-inflammatory efficacy between CTS and MC (*p* < 0.001 versus individual components).

#### 3.3.4. Cytokine Modulation in RAW264.7 Cells

CTS and MC synergistically inhibit LPS-induced cytokine release in RAW264.7 macrophages. LPS (1 μg/mL) significantly elevated TNF-α, IL-1β, IL-6, and PGE_2_ versus controls (*p* < 0.001 vs. control). Positive control (DG, 50 μM) suppressed all cytokines to near-baseline (*p* < 0.001 vs. LPS). CTS (0.5 μg/mL) reduced TNF-α/IL-1β/PGE_2_ (*p* < 0.01) and IL-6 (*p* < 0.05 vs. LPS). MC (100 μg/mL) potently inhibited TNF-α/IL-1β/IL-6 (*p* < 0.01 vs. LPS) and moderately suppressed PGE_2_ (*p* < 0.05 vs. LPS).

**Figure 6:**
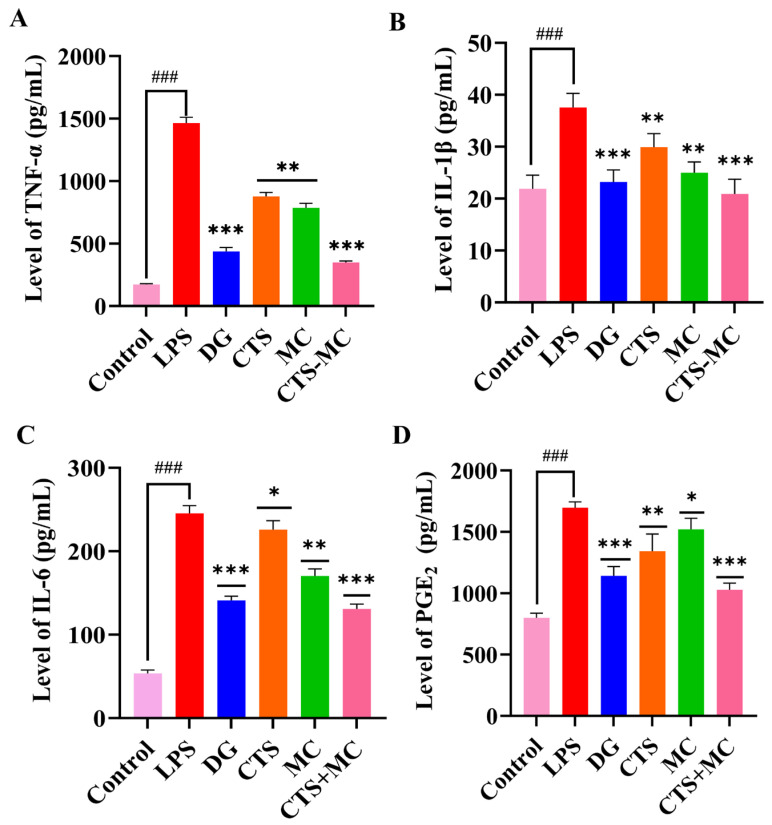
ELISA quantification in RAW264.7 cells. (**A**) TNF-α, (**B**) IL-1β, (**C**) IL-6, and (**D**) PGE_2_ levels. Data were presented as mean ± SEM. “###” denoted *p* < 0.001 compared with the blank control. Wherever included, comparisons with the model group were indicated as * *p* < 0.05, ** *p* < 0.01, and *** *p* < 0.001.

The combination of MC (100 μg/mL) and CTS (0.5 μg/mL) demonstrated synergistic efficacy, reducing all cytokines to 20–40% of LPS levels (*p* < 0.001 vs. LPS), comparable to DG. Mechanistic analysis revealed complementary targeting: CTS preferentially modulated TNF-α/IL-1β/PGE_2_ while MC selectively suppressed TNF-α/IL-1β/IL-6 cascades (Figure 6).

### 3.4. Rat Acne Model and Therapeutic Intervention

#### 3.4.1. Therapeutic Efficacy in Acne Model

**Figure 7:**
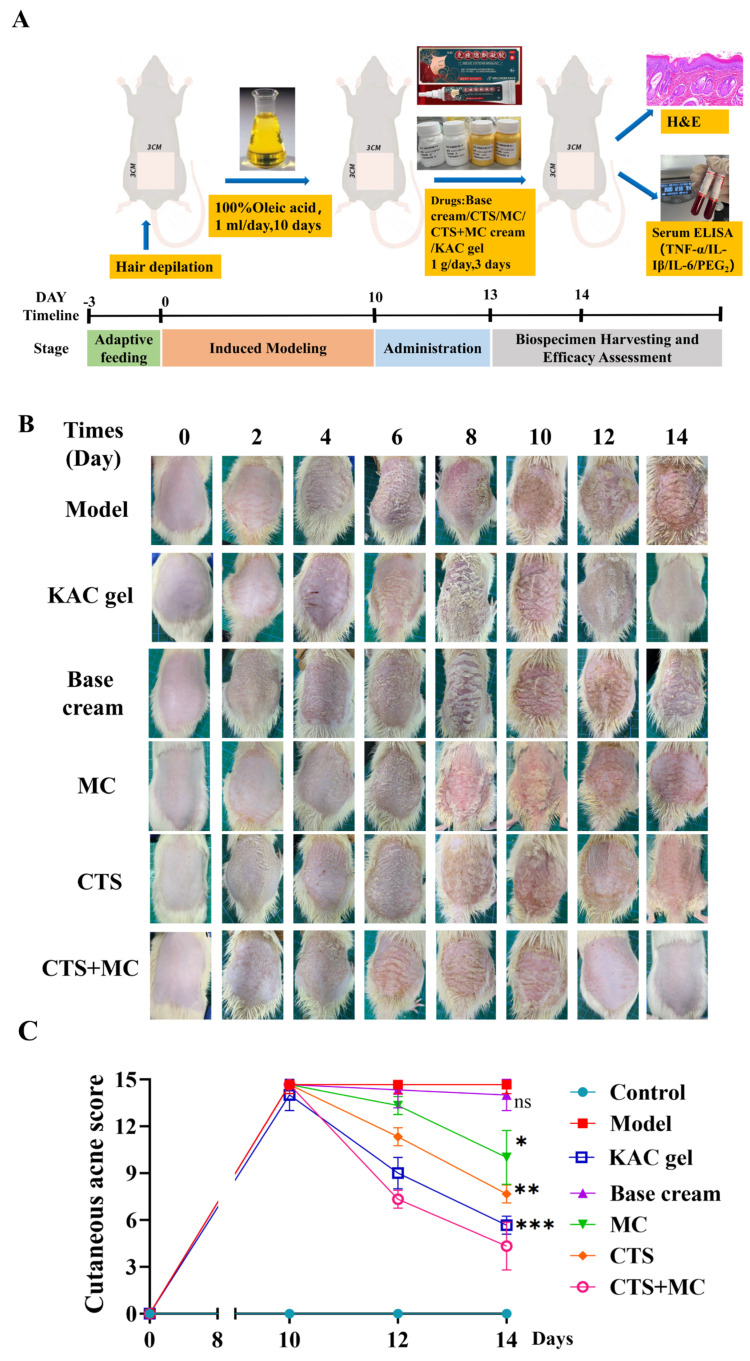
Therapeutic evaluation in an acne model. (**A**) Schematic diagram of SD rat treatment; (**B**) Macroscopic skin lesions in SD rats; (**C**) Cutaneous acne severity scores. Data were presented as mean ± SEM. comparisons with the model group were indicated as n.s. (non-significant, *p* > 0.05), * *p* < 0.05, ** *p* < 0.01, and *** *p* < 0.001.

In the oleic acid-induced rat acne model, characteristic pathological manifestations emerged beginning on Day 5 of modelling, characterized by significant stratum corneum hyperplasia, enhanced epidermal scaling, and progressive local erythema (Figure 7A,B). Pathological severity peaked at Day 10 (Figure 7C); modelling was subsequently terminated, and therapeutic intervention was initiated (1 g/application).

On Day 12, all intervention groups except the model group exhibited early improvement trends, including reduced epidermal erythema and decreased scaling coverage. Final assessment at Day 14 revealed that the model group maintained severe acne characteristics with persistent hyperkeratosis, surface roughness, plaque-like desquamation, and elevated cutaneous acne scores. The base cream group demonstrated no significant anti-acne activity (*p* > 0.05 vs. the model); and the KAC gel group displayed rapid anti-inflammatory and keratin-regulatory effects, with the cutaneous phenotype approaching normalization and significantly reduced cutaneous acne scores (*p* < 0.001 vs. model).

In the monotherapy groups, MC and CTS significantly lowered cutaneous acne scores (*p* < 0.05 vs. the model), and CTS treatment yielded greater skin smoothness than MC. The CTS + MC combination group achieved complete cutaneous normalization (full resolution of erythema and scales), demonstrating superior efficacy versus monotherapies (*p* < 0.001 vs. model).

#### 3.4.2. H&E

**Figure 8:**
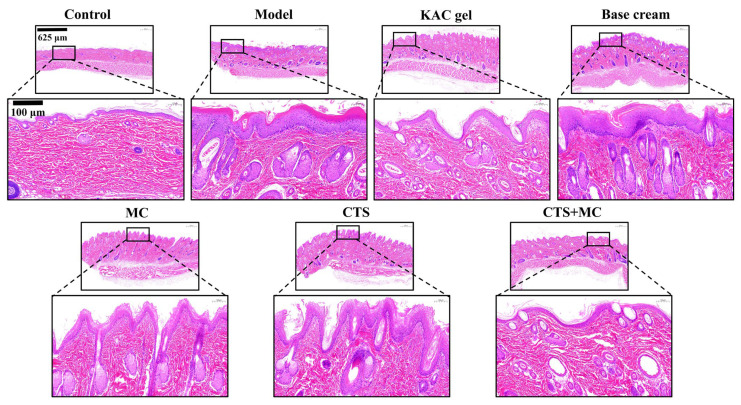
Histopathology determined by H&E staining.

Histopathological analysis revealed severe epidermal hyperplasia (45.2 ± 3.1 μm), pronounced follicular hyperkeratosis with keratotic plugs, and dense dermal inflammatory infiltration in the model group (Figure 8). MC monotherapy significantly reduced epidermal thickness to 28.5 ± 2.3 μm (*p* < 0.01 vs. the model), decreased follicular hyperkeratosis by 68% (*p* < 0.01), and reduced the inflammatory cell count to 42 ± 8 cells/HPF (*p* < 0.01 vs. model). CTS monotherapy demonstrated moderate improvement: epidermal thickness 32.7 ± 2.9 μm (*p* < 0.05 vs. model), 39% reduction in hyperkeratosis (*p* < 0.05 vs. model), and 65 ± 10 inflammatory cells/HPF (*p* < 0.05 vs. model). The CTS + MC combination achieved near-complete normalization: epidermal thickness restored to 18.3 ± 1.7 μm (comparable to the control), a 94% reduction in follicular hyperkeratosis (*p* < 0.001 vs. model), and inflammation was reduced to 18 ± 5 cells/HPF (*p* < 0.001 vs. model; n.s. vs. control). The positive control (KAC gel) significantly reduced epidermal thickness to 20.1 ± 1.9 μm and inflammatory cells to 25 ± 6 cells/HPF (*p* < 0.01 vs. model for both parameters), while the base cream demonstrated no significant improvements versus the model (*p >* 0.05).

#### 3.4.3. ELISA of Serum

An ELISA analysis of inflammatory markers revealed a statistically significant elevation in cytokine levels within the model group compared to baseline controls. Treatment with the base cream formulation demonstrated no statistically significant effect on the elevated cytokine levels relative to the model group, effectively excluding any substantial contribution from the vehicle itself. In contrast, the positive control group (KAC gel) exhibited a statistically significant reduction in all measured inflammatory cytokines compared to the model group (*p* < 0.001).

**Figure 9:**
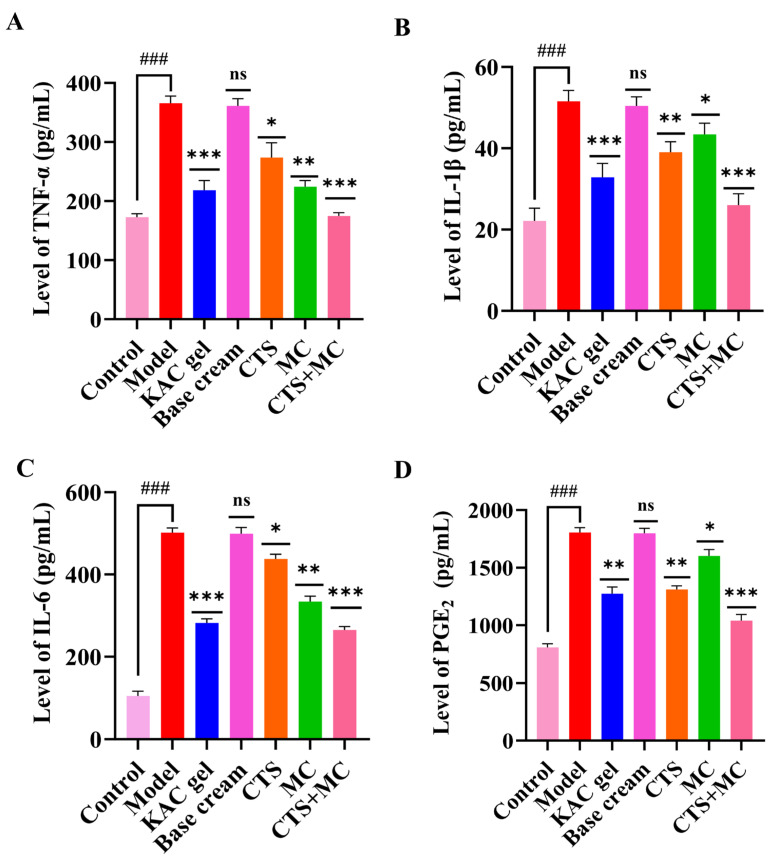
Serum inflammatory cytokines associated with ELISA. (**A**) TNF-α, (**B**) IL-1β, (**C**) IL-6, and (**D**) PGE_2_ levels. Data were presented as mean ± SEM. “###” denoted *p* < 0.001 compared with the blank control. Wherever included, comparisons with the model group were indicated as n.s. (non-significant, *p* > 0.05), * *p* < 0.05, ** *p* < 0.01, and *** *p* < 0.001.

Both MC monotherapy and CTS monotherapy demonstrated inhibitory effects on the levels of IL-1β, IL-6, and PGE_2_. However, the magnitude of inhibition achieved by either monotherapy was consistently less pronounced than that observed with the KAC gel positive control. (*p* < 0.05 vs. model). Crucially, the combination therapy of MC and CTS resulted in a significantly enhanced anti-inflammatory effect compared to either monotherapy administered alone. The efficacy of the combination therapy approached the level of inhibition observed in the KAC gel positive control group (*p* < 0.001 vs. model; n.s. vs. KAC gel) (Figure 9).

## 4. Discussion

### 4.1. Main Research Findings

Acne vulgaris arises from the interplay of genetic, endocrine, microbial, and immune factors [27,28,29]. Current therapies face challenges such as antimicrobial resistance and side effects, driving the search for safer, multi-target alternatives derived from natural products [30]. In this study, we investigated the combined use of CTS and MC—two plant-derived bioactive compounds—for the management of acne. Our key findings demonstrate that the CTS–MC combination exhibits enhanced efficacy and reduced toxicity compared to either compound alone, highlighting its potential as a promising anti-acne agent.

CTS has long been used to improve microcirculation, reduce inflammation, and repair skin lesions. Clinically, it is applied in formulations for inflammatory and pustular acne (e.g., OTC creams containing CTS). Its antimicrobial activity extends to *S. aureus*, *S. epidermidis*, and *C. acnes* [28], potentially via disruption of bacterial respiration (inhibition of NADH dehydrogenases) and induction of ROS [29]. CTS also exerts anti- and anti-androgenic effects [6]. However, its irritancy risk at high local concentrations prompted the development of delivery systems such as CTS–peptide–conjugated nanoparticles, glycyrrhizic acid (GA)-CTS micelles, and ceramide-coated liposomes, which improve loading, release, and stability [30]. Nonetheless, these systems may suffer structural instability under processing or storage, limiting their scalability and widespread market application [31,32]. Moreover, despite exhibiting nanoscale architecture, CTS nano-liposomes are excluded from the EU regulatory definition of nanomaterials due to their inherent biodegradability and solubility. It is important to note, however, that their innovative status as delivery vehicles necessitates a rigorous safety assessment to guarantee regulatory compliance and operational safety.

MC, the major bioactive component of *Centella asiatica* complements CTS by promoting fibroblast regeneration, dermal–epidermal junction integrity, and ECM remodelling [23]. It downregulates TNF-α, IL-6, PGE_2_, and COX-2 and is widely incorporated into anti-acne and soothing skin-care formulations [25,33,34]. Notably, combining MC directly with CTS avoids nanomaterial-related safety and regulatory concerns.

Zebrafish provide a translational in vivo model (82% genetic homology with humans) and were used here to assess inflammation, toxicity, and wound-healing endpoints [13]. In our experiments, we observed that 24 hpf zebrafish embryos self-rotate at a baseline frequency, with increased rotation upon exposure to irritants [35], supporting their use for cosmetic irritancy evaluation.

Our results demonstrate that while CTS alone exhibits potent antimicrobial and anti-inflammatory effects (MIC 62.5 μg/mL, inhibition zone 1.5× positive control Q-73), toxicity and irritancy limit its application. MC alone demonstrated minimal antibacterial activity. Remarkably, the CTS–MC combination significantly reduced zebrafish mortality and irritancy while maintaining antimicrobial and anti-inflammatory activity, suggesting a synergistic interaction via multifunctional mechanisms: (1) CTS rapidly kills *C. acnes* and reduces inflammatory triggers; (2) MC attenuates CTS-induced toxicity and enhances tissue repair; (3) both compounds contribute to immune–microbiota homeostasis. This direct combination offers an effective strategy while avoiding the regulatory hurdles associated with nano-enabled systems.

This synergistic effect was further validated in a mammalian model. In the oleic acid-induced rat acne model, topical CTS–MC cream solubilized in DPG with sodium surfactin emulsifier (which was derived via biological fermentation) achieved superior efficacy compared with either compound alone, approaching that of the positive control (KAC gel). Improvements included reduced epidermal hyperplasia, decreased follicular keratinization, attenuated neutrophil infiltration, and smaller sebaceous glands. Formulation advantages included improved solubility, stability, skin adherence, and reduced irritancy.

Importantly, we employed high-purity CTS (≥98%) and MC (≥95%), avoiding the variability of crude extracts. Compared with synthetic drugs, the CTS–MC combination offers mildness and resistance-proof activity and, compared with single-herb TCM, greater efficacy.

### 4.2. Limitations and Future Directions

The selection of DPG as a solvent was based on preliminary solubility and stability data; however, this study did not compare DPG with other solubilizing agents in Franz cell assays, and the pharmacokinetics of CTS and MC alone or in combination remain uncharacterized. Although the multi-target anti-acne effects were confirmed, detailed mechanisms relating to hyperkeratinization, sebaceous regulation, and androgen modulation require further investigation.

The toxicological data underscores the narrow biocompatibility window of CTS; Phase I clinical trials are needed to establish safety and tolerability in humans. Future work should also focus on optimizing delivery systems to enhance stability and reduce potential side effects.

## 5. Conclusions

This study demonstrates that the synergistic combination of CTS and MC effectively targets acne pathogenesis through complementary mechanisms. In vitro, CTS exhibited potent antibacterial activity against *C. acnes* and suppressed pro-inflammatory cytokines, while MC attenuated CTS-induced cytotoxicity in keratinocytes and further modulated macrophage inflammatory responses. In animal models (zebrafish and rat), the combination of CTS + MC simultaneously inhibited neutrophil migration and inflammation, reduced follicular hyperkeratosis, and accelerated barrier repair without causing significant skin irritancy. The optimized topical formulation supports translational applicability for acne therapy. This functional complementarity strategy represents a promising paradigm for developing safer multi-target treatments that leverage natural compounds. Future work should validate efficacy in clinical settings and explore its impacts on the dysregulation of the microbiome–lipid axis.

## Figures and Tables

**Table 1:** Treatment groups for the cytotoxicity assay in HaCaT cells.

Group	Compounds	Concentration Range
CTS	CTS alone	0–50 μg/mL + 1% DPG
MC	MC alone	0–2000 μg/mL + 1% DPG
CTS + MC	CTS + MC	CTS: 0–2000 μg/mL; MC fixed at 500 μg/mL +1% DPG
Control	Culture medium only	1% DPG

**Table 2:** Treatment groups for inflammatory cytokine induction in RAW 264.7 cells.

Group	Treatment Description	Concentration Range
Control	DMEM-H medium	/
Model	LPS	1 μg/mL
Positive control	LPS + dipotassium glycyrrhizate	1 μg/mL of LPS; 50 μM of dipotassium glycyrrhizate
MC	LPS + MC	1 μg/mL of LPS; 100 μg/mL of MC
CTS	LPS + CTS	1 μg/mL of LPS; 0.5 μg/mL of CTS
CTS + MC	LPS + CTS + MC	1 μg/mL of LPS; MC (100 μg/mL) + CTS (0.5 μg/mL)

## Data Availability

The raw data supporting the conclusions of this article will be made available by the authors on request.

## Abbreviations

The following abbreviations are used in this manuscript:

ANOVA	Analysis of Variance
*C. acnes*	*Cutibacterium acnes* (Formerly *Propionibacterium acnes*)
CCK-8	Cell Counting Kit-8
COX-2	Cyclooxygenase-2
CTS	Cryptotanshinone
DEX	Dexamethasone
DG	Dipotassium Glycyrrhizinate
DMEM	Dulbecco’s Modified Eagle Medium
DMSO	Dimethyl Sulfoxide
DPG	Dipropylene Glycol
dpf	Days Post-fertilization (Zebrafish Developmental Stage)
ECM	Extracellular Matrix
ELISA	Enzyme-Linked Immunosorbent Assay
EWG	Environmental Working Group
FBS	Fetal Bovine Serum
GA	Glycyrrhizic acid
GTCC	Caprylic/Capric Triglyceride
hpf	H Post-fertilization (Zebrafish Developmental Stage)
H&E	Hematoxylin–Eosin (Staining)
HPF	High-Power Field
IC_50_	Half-Maximal Inhibitory Concentration
IL	Interleukin
LC_50_	Lethal Concentration 50
LPS	Lipopolysaccharide
MC	Madecassoside
MIC	Minimum Inhibitory Concentration
NADH	Nicotinamide Adenine Dinucleotide (reduced form)
NF-κB	Nuclear Factor Kappa-light-chain-enhancer of Activated B cells
OD	Optical Density
PBS	Phosphate-Buffered Saline
PGE_2_	Prostaglandin E_2_
RCM	Reinforced Clostridial Medium
ROS	Reactive Oxygen Species
SD rats	Sprague–Dawley Rats
SDS	Sodium dodecyl sulfate
SEM	Standard Error of the Mean
*S. miltiorrhiza*	*Salvia miltiorrhiza*
TLR_2_	Toll-like Receptor _2_
TNF-α	Tumor Necrosis Factor-alpha
WT	Wild-Type
